# Pharmacogenetic Variants in the *DPYD* and *TYMS* Genes are Clinically Significant Predictors of Fluoropyrimidine Toxicity: Are We Ready for Use in our Clinical Practice

**Published:** 2020

**Authors:** Muhammad Wasif Saif, Hilal Hachem, Sneha Purvey, Ruchi Hamal, Lulu Zhang, Nauman Saleem Siddiqui, Amandeep Godara, Robert B. Diasio

**Affiliations:** 1 Northwell Health Cancer Institute, Lake Success, NY 11042, USA; 2 Tufts Medical Center, Boston, MA, 02110, USA; 3 Mayo Clinic, Rochester, MN, Rochester, MN, USA

**Keywords:** 5-Fluorouracil, Capecitabine, Fluoropyrimidines, DPYD gene

Fluoropyrimidines have been extensively used for almost 6 decades to treat a variety of solid cancers, especially colon, gastric, anal, rectal, head & neck and breast [1–4] (Table 1).

However, 31–34% of patients encountered grade 3–4 adverse events (AEs) with 0.5% mortality often-necessitating dose reduction or discontinuation [5]. A significant proportion of these AEs are likely to be the result of inter-individual genetic variation, in particularly such as dihydropyrimidine dehydrogenase (*DPYD*). *DPYD* gene encodes DPD, the rate-limiting enzyme responsible for catabolism of 5-FU and is responsible for >85% of 5-FU elimination. Deficiency of *DPD* due to DPYD polymorphism gives rise to severe 5-FU AEs from reduced catabolism [6]. This pharmacogenetic ‘*DPD syndrome*’ manifests typically as severe or fatal diarrhea, mucositis/stomatitis, myelosuppression and even rare toxicities, such as hepatitis, encephalopathy and acute cardiac ischemia following first or second dose of 5-FU [6–8]. *DPYD* mutations are found in 50% of severe 5-FU toxicity cases [6–10]. Different methods have been developed to test *DPYD* abnormalities [11,12].

In addition to DPYD, other pharmacogenetic markers, such as thymidylate synthase (*TYMS*) has also been reported but with conflicting results [13]. *TYMS* catalyzes methylation of dUMP to dTMP. As the sole *de novo* source of thymidylate in the cell it is an important target for 5-flurouracil (5-FU) and capecitabine (CAP). Overexpression of *TYMS* has been linked to resistance to these agents both *in vitro* and *in vivo*. Cause of variability in *TYMS* expression is unclear, however, polymorphisms in 5 and 3 untranslated regions (5UTR and 3UTR) of *TYMS* gene have been described previously and these are suggested to be both predictive to toxicity and prognostic to efficacy with fluoropyrimidines [13–18]. However, the data remains unsettled at present.

Despite the richness of data and constant concern about potential toxicity, especially in relation to *DPYD* no pharmacogenetic markers of fluoropyrimidine AEs have been recommended by any agencies or organizations till 13 March 2020, the European Medicines Agency’s (EMA’s) Pharmacovigilance Risk Assessment Committee (PRAC) has recommended that patients receiving fluorouracil given by injection or infusion and the related medicines capecitabine and tegafur should be tested for the lack of DPD before starting treatment [19]. The general guidelines re summarized in Table 2. PRAC has allowed both methods of testing, including measuring the level of uracil in the blood, or by checking for the presence of certain mutations in the gene for DPD which are associated with an increased risk of severe side effects [19].

The committee did not mandate the pre-treatment testing or dose adjustments based on DPD activity for patients using topical fluorouracil as the level of fluorouracil absorbed through the skin into the body is extremely low, and the safety of topical fluorouracil is not expected to change in patients with partial or complete DPD deficiency [19].

Our group has also persistently studying the pharmacogenetic markers associated with these cytotoxic drugs. Here, we described a summary of our study that aimed to identify pharmacogenetic markers predicting fluoropyrimidine AEs. We recorded AEs following 5-FU or capecitabine in a series of 430 patients to associate with *DPYD* and *TYMS*. A total of 52 patients were identified with *DPYD* abnormalities: 11/12 patients had low *DPYD* activity (range: 0.064 −0.18 nmol /min/ mg). *DPYD* genotyping showed: IVS14 + 1 G > A (c.1905+1 G > A, rs3918290) 38%, D949V (C.2846A > T, rs67376798) 21%, C29R (rs1801205) 4%, and Y186C (rs115232898, c-557 A > G) 2%. UraBT confirmed *DPD* deficiency in 2 patients: DOB_50_ of 49.4% and 52.5%. *TYMS* genotype abnormalities were identified in 38 patients including 2 patients with both *TYMS* and *DPYD* abnormalities. Distributions for *TYMS* abnormalities were: 5’-TSER: 53% with low expression genotypes (10: 2R/2R; 21: 2R/3RC; 23: 3RC/3RC) and 47% with high expression genotypes (11: 2R/3RG, 54: 3RG/3RC, 37: 3RG/3RG) and 3’-UTR were: 18% with INS/INS (normal), 45% INS/ DEL (intermediate) and 13.6% DEL/DEL (low). 68.7% of patients have ≥ 1 abnormality. All *DPYD* sequence variants and *TYMS* del/del or dual abnormalities of 5’-TSER/3’-UTR were significantly associated with grade 3–4 AEs.

Our data clearly supports the decision made by EMA’s PRAC. Recently, uridine triacetate (Vistogard) was approved by FDA to help cancer patients who developed severe toxicity to fluoropyrimidines or overdose [20,21].

We may like to add that probably combined *DPYD* and *TYMS* genotyping could identify ≥ 50% of patients, who are at greatest risk of AEs. At present, no formal recommendations regarding testing for *DPYD* exist in USA except warning on FDA website and prescription inserts. We hope prospective studies will validate the role of *TYMs* and that *DPYD* will also be adopted soon in USA.

## Figures and Tables

**Table 1:** Indications for 5-Fluorouracil.

• Colon and rectal cancer
• Anal cancer
• Breast cancer
• Esophageal cancer
• Pancreatic cancer
• Gastric cancer
• Head and neck cancer
• Carcinoma of unknown primary (esp. squamous cell histology)
• Neuroendocrine tumors
• Thymic cancers
• Cervical cancer
• Bladder cancer
• Hepatobiliary cancers
• Topical 5-FU in basal cell cancer of the skin and actinic keratoses

**Table 2:** Dose recommendation in patients with DPYD abnormalities.

DPD Deficiency	Risk of AEs	Recommendations
Complete DPD deficiency	Higher risk of severe and life-threatening	• Must not administer fluorouracil injection or infusion, capecitabine or tegafur.
Partial DPD deficiency	Increased risk but variable	•Start these drugs at reduced starting dose. • If tolerated, the dose may be increased if there are no serious side effects as the effectiveness of a reduced dose has not been established. • Regular monitoring of drug levels in the blood may help in certain patients.
